# PLA_2_R Antibody Levels and Clinical Outcome in Patients with Membranous Nephropathy and Non-Nephrotic Range Proteinuria under Treatment with Inhibitors of the Renin-Angiotensin System

**DOI:** 10.1371/journal.pone.0110681

**Published:** 2014-10-14

**Authors:** Elion Hoxha, Sigrid Harendza, Hans Pinnschmidt, Ulf Panzer, Rolf A. K. Stahl

**Affiliations:** 1 III. Medizinische Klinik, Universitätsklinikum Hamburg-Eppendorf, Hamburg, Germany; 2 Institut für Medizinische Biometrie & Epidemiologie, Universitätsklinikum Hamburg-Eppendorf, Hamburg, Germany; University Medical Center Groningen and University of Groningen, Netherlands

## Abstract

Patients with primary membranous nephropathy (MN) who experience spontaneous remission of proteinuria generally have an excellent outcome without need of immunosuppressive therapy. It is, however, unclear whether non-nephrotic proteinuria at the time of diagnosis is also associated with good prognosis since a reasonable number of these patients develop nephrotic syndrome despite blockade of the renin-angiotensin system. No clinical or laboratory parameters are available, which allow the assessment of risk for development of nephrotic proteinuria. Phospholipase A_2_ Receptor antibodies (PLA_2_R-Ab) play a prominent role in the pathogenesis of primary MN and are associated with persistence of nephrotic proteinuria. In this study we analysed whether PLA_2_R-Ab levels might predict development of nephrotic syndrome and the clinical outcome in 33 patients with biopsy-proven primary MN and non-nephrotic proteinuria under treatment with blockers of the renin-angiotensin system. PLA_2_R-Ab levels, proteinuria and serum creatinine were measured every three months. Nephrotic-range proteinuria developed in 18 (55%) patients. At study start (1.2±1.5 months after renal biopsy and time of diagnosis), 16 (48%) patients were positive for PLA_2_R-Ab. A multivariate analysis showed that PLA_2_R-Ab levels were associated with an increased risk for development of nephrotic proteinuria (HR = 3.66; 95%CI: 1.39–9.64; p = 0.009). Immunosuppressive therapy was initiated more frequently in PLA_2_R-Ab positive patients (13 of 16 patients, 81%) compared to PLA_2_R-Ab negative patients (2 of 17 patients, 12%). PLA_2_R-Ab levels are associated with higher risk for development of nephrotic-range proteinuria in this cohort of non-nephrotic patients at the time of diagnosis and should be closely monitored in the clinical management.

## Introduction

Membranous nephropathy (MN) is the most common cause of nephrotic syndrome in adults. The clinical outcome is variable and ranges from spontaneous remission of proteinuria to end stage renal failure. Statistical models have delineated elevated serum creatinine, male gender, hypertension, older age and high proteinuria as predictors of poor renal outcome [1]. Even though high level of proteinuria at the time of diagnosis represents a risk for loss of renal function, long term follow-up studies have shown that spontaneous remission of nephrotic range proteinuria is a frequent event in patients with MN. Patients with spontaneous remission of proteinuria have an excellent long term renal prognosis and do not need immunosuppressive therapy [2]. Similarly, patients with non-nephrotic proteinuria at onset of the disease, which persists during the follow-up, also have a good long-term prognosis [3]. However, non-nephrotic proteinuria at the time of diagnosis does not always indicate a good prognosis since nephrotic syndrome may develop during the course of the disease [3]. The course of the disease can only be detected by follow-up measurements of proteinuria in these patients. PLA_2_R-Ab are present in many patients with primary MN and high antibody levels are associated with a longer persistence of nephrotic range proteinuria during treatment [4], [5]. We therefore prospectively analysed whether PLA_2_R-Ab levels at the time of diagnosis of primary MN are associated with the long-term clinical outcome of patients with non-nephrotic range proteinuria already treated with inhibitors of the renin-angiotensin system.

## Materials and Methods

### Patients and study design

Inclusion criteria for participating in this prospective multicenter open clinical study were histologic diagnosis of primary MN, a serum test for PLA_2_R-Ab within six months of renal biopsy, proteinuria of <3.5 g/24 h and no immunosuppressive therapy prior to inclusion in the study. Secondary MN led to the exclusion of patients from the study. Patients were screened by the treating physicians. This included serologic tests for lupus erythematodes, hepatitis, a detailed medical history and a screening for malignancies depending on the age and risk factors of the patient. During the study follow-up the treating physicians decided on the therapeutic strategy without any recommendations. However, the most common factors for starting immunosuppressive treatments were severe clinical symptoms such as nephrotic syndrome and edema not responding to supportive therapy. A further parameter for start of immunosuppression was the decline of renal function. Since the role of PLA_2_R-Ab levels in predicting the development of disease or treatment success was unclear at the beginning of the study, all treatment decisions were based on the clinical experience of the treating physicians and not the PLA_2_R-Ab levels.

PLA_2_R-Ab levels, 24-hour protein excretion and serum creatinine were measured every three months. The study was approved by the local ethics committee of the chamber of physicians in Hamburg and conducted in accordance with the ethical principles stated by the Declaration of Helsinki. Written informed consent was obtained from all participating patients.

### Measurement of proteinuria, serum albumin and PLA_2_R-Ab levels

Serum levels of PLA_2_R-Ab were measured by an ELISA which was published and validated earlier [6]. According to the manufacturer, the ELISA results were considered positive at a level >20 U/ml. Proteinuria is given as total 24-hour excretion and serum creatinine in mg/dl. In patients who developed nephrotic-range proteinuria during the study follow-up, remission of proteinuria was defined as proteinuria of <3.5 g/24 h and at least 50% reduction from the highest level of proteinuria prior to remission, complete remission of proteinuria was defined as proteinuria <0.5 g/24 h. A significant increase of serum creatinine was defined as an increase by ≥25% compared to the time of study inclusion and a serum creatinine ≥1.3 mg/dl.

### Statistical analysis

Data are given as mean values ± SD. Statistical significance was defined as p<0.05 (α = 0.05). Survival analysis was done on the endpoint defined as development of nephrotic range proteinuria (proteinuria >3.5 g/24 h) by computing Kaplan-Meier curves for PLA_2_R-Ab positive and negative patients. The Kaplan-Meier curves were compared by log rank tests for significant differences. A multivariate Cox regression analysis was performed on the endpoint development of nephrotic range proteinuria, employing clinical variables measured at the beginning of the study (age, sex, total IgG PLA_2_R-Ab levels, proteinuria) as explanatory variables. The explanatory variable PLA_2_R-Ab levels were ln-transformed prior to this analysis. The strength and significance of effects of the explanatory variables were indicated by hazard ratios and their associated confidence intervals and p values in a forest plot. All statistical analyses were done using SPSS version 21.

## Results

### Clinical Baseline Characteristics

Thirty-three patients with biopsy proven primary MN and proteinuria <3.5 g/24 h were included in this study (Table 1). The time between renal biopsy and study inclusion (first measurement of PLA_2_R-Ab) was 1.2±1.5 months. Patients were prospectively followed for 25.3±8.8 months after recruitment. At the time of study start 30 (91%) patients were treated with an ACE-inhibitor or angiotensin ll-receptor-blocker, 20 (61%) patients received diuretics, 15 (45%) patients received statins and five (15%) patients received anticoagulants, although they had non-nephrotic proteinuria. Two patients were put on treatment with anticoagulants after thromboembolic events before inclusion in the study (one renal vein thrombosis and one lung artery embolism). A third patient had proteinuria of 1.8 g/24 h, but a serum albumin of 19 g/l, which resulted in the treatment with anticoagulants. The additional two patients were treated with anticoagulants because of unrelated conditions.

**Table 1:** Clinical baseline characteristics of the patients at the time of inclusion in the study.

	Wholepatientcohort	PLA_2_R-Abnegative	PLA_2_R-Abpositive	P-value
Number of Patients	33	17	16	
Sex (m/w - %male)	20/13–61%	9/8–53%	11/5–69%	0.481
Age (years)	50.1±19.2	51.8±21.7	48.3±16.6	0.600
Time from renal biopsy tofirst serum measurement (months)	1.2±1.5	1.6±1.7	0.7±1.0	0.074
Proteinuria (mg/24 h)	2031±876	1774±958	2304±708	0.082
Serum creatinine (mg/dl)	1.0±0.5	1.1±0.5	1.0±0.4	0.425
PLA_2_R-Ab level (U/ml)	194±545	3.7±3.2	396±741	0.037
Patients on immunosuppressivetherapy during follow-up (%)	15 (45%)	2 (12%)	13 (81%)	<0.001
Follow-up time in the study (months)	25.3±8.8	26.1±9.5	24.4±8.3	0.578

At the time of inclusion in the study 16 (48%) patients had detectable PLA_2_R-Ab in the serum. Proteinuria at inclusion in the study was higher in PLA_2_R-Ab positive patients compared to PLA_2_R-Ab negative patients but this difference did not reach statistical significance. Serum creatinine and age were not different between PLA_2_R-Ab positive and PLA_2_R-Ab negative patients. Significantly more PLA_2_R-Ab positive patients received immunosuppressive treatment during the follow-up time.

**Figure 1:**
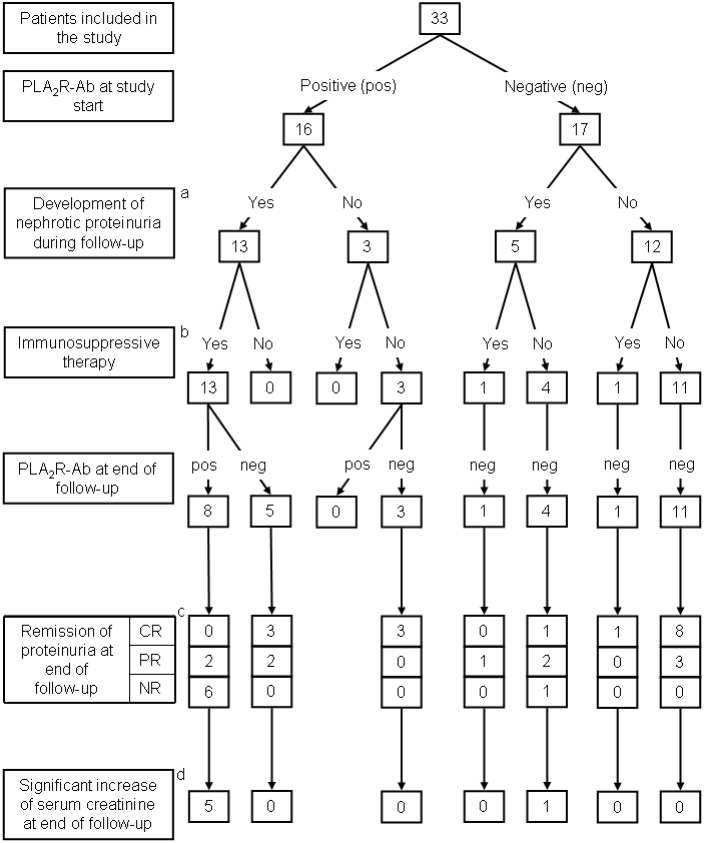
Flow chart of patients included in the study and their follow-up. Of the 33 patients included in the study 16 were PLA_2_R-Ab positive and 17 were PLA_2_R-Ab negative at the start of the study. Nephrotic proteinuria developed in 13 PLA_2_R-Ab positive and five PLA_2_R-Ab negative patients. Immunosuppressive treatment was used in 13 PLA_2_R-Ab positive patients and two PLA_2_R-Ab negative patients. At the end of the follow-up eight patients were still PLA_2_R-Ab positive, two of them had a remission of proteinuria (both partial remission) and five of them had a significant increase in serum creatinine. PLA_2_R-Ab were negative in 25 patients at the end of the follow-up, 24 of them had a remission of proteinuria (16 complete remission, eight partial remission) and one of them had a significant increase in serum creatinine. CR = complete remission; PR = partial remission; NR = no remission. ^a^ = Significantly more PLA_2_R-Ab positive patients developed nephrotic-range proteinuria compared to PLA_2_R-Ab negative patients (Fisher’s exact test: p<0.005). ^b^ = Significantly more PLA_2_R-Ab positive patients received immunosuppressive therapy compared to PLA_2_R-Ab negative patients (Fisher’s exact test: p<0.001). ^c^ = Significantly less patients who were still positive for PLA_2_R-Ab at the end of the study follow-up reached remission of proteinuria compared to patients who were negative for PLA_2_R-Ab at the end of the follow-up (Fisher’s exact test: p<0.001). ^d^ = Significantly more patients who were still positive for PLA_2_R-Ab at the end of the study follow-up had a significant increase of serum creatinine compared to patients who were negative for PLA_2_R-Ab at the end of the follow-up (Fisher’s exact test: p = 0.001).

During the follow-up of the study all 33 patients received an ACE-inhibitor or an angiotensin-receptor-blocker. Fifteen (45%) patients received an immunosuppressive treatment during the study follow-up. At study start, 17 (52%) of the 33 patients were negative for PLA_2_R-Ab in the serum, 16 (48%) patients were positive for PLA_2_R-Ab (Figure 1).

Data on the PLA_2_R staining of biopsy specimens are available from eight of the 17 PLA_2_R-Ab negative patients. In three of these biopsy specimens an enhanced PLA_2_R staining could be detected, five patients showed no enhancement for PLA_2_R staining.

### Development of nephrotic-range proteinuria during follow-up

**Figure 2:**
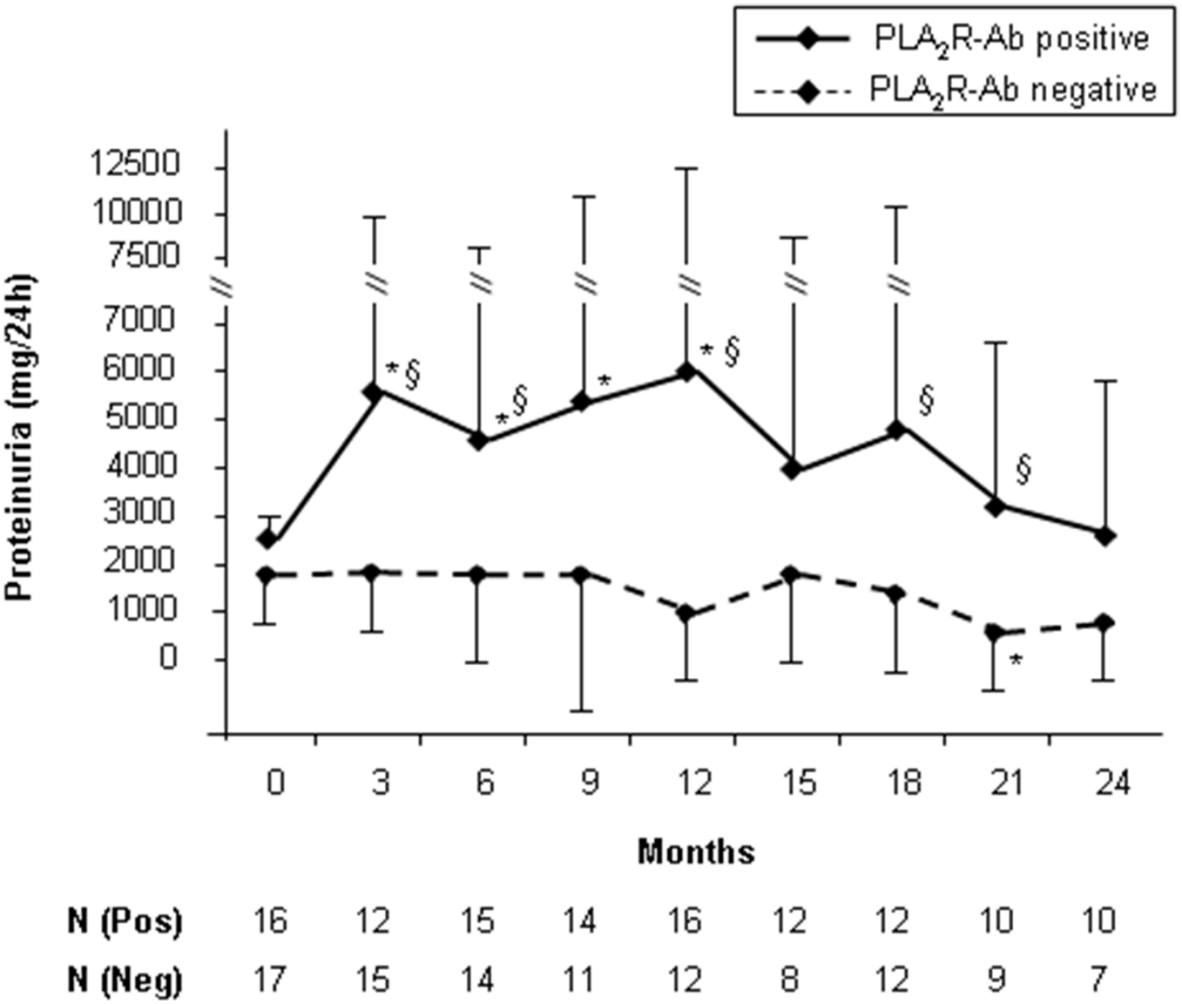
Proteinuria during the study follow-up. Proteinuria significantly increased in PLA_2_R-Ab positive patients (solid line) but remained low in PLA_2_R-Ab negative patients (dashed line). The bars show the SD-values of proteinuria for PLA_2_R-Ab positive patients (up) and PLA_2_R-Ab negative patients (down). “N” gives the number of patients for whom data were available at these specific times of follow-up. “*” shows a statistically significant difference (p<0.05) between the single time point and the start of the study. “§” shows a statistically significant difference (p<0.05) between PLA_2_R-Ab positive patients and PLA_2_R-Ab negative patients.

Within three months after inclusion in the study, proteinuria increased from 2.0±0.9 g/24 h to 3.4±3.3 g/24 h in the whole study cohort. This increase was due to the 138% increase in proteinuria in PLA_2_R-Ab positive patients in the first three months of the study (Figure 2). At the same time, in PLA_2_R-Ab negative patients proteinuria remained stable at 1.8±1.0 g/24 h. At every single time point during the study proteinuria was higher in PLA_2_R-Ab positive patients compared to PLA_2_R-Ab negative patients.

During the study observation, 18 (55%) patients developed nephrotic-range proteinuria (Figure 1). When considering the PLA_2_R-Ab levels at the start of the study, significantly more PLA_2_R-Ab positive patients developed nephrotic-range proteinuria (13 out of 16) compared to PLA_2_R-Ab negative patients (five out of 17) (Fishers exact test: p<0.005). At the same time, the 13 PLA_2_R-Ab positive patients developed a more severe nephrotic syndrome with highest proteinuria levels reaching 10.4±5.7 g/24 h, compared to 5.4±1.9 g/24 h in the 5 PLA_2_R-Ab negative patients (p = 0.08).

All three patients with enhanced PLA_2_R staining in the biopsy and negative PLA_2_R-Ab remained negative for PLA_2_R-Ab during the study follow-up. Two of them did not develop nephrotic range proteinuria and had a complete remission of proteinuria at the end of the study follow-up. The third patient developed nephrotic range proteinuria, but had a spontaneous remission of proteinuria at the end of the study follow-up.

### Analysis of PLA_2_R-Ab levels as risk factor for development of nephrotic proteinuria

**Figure 3:**
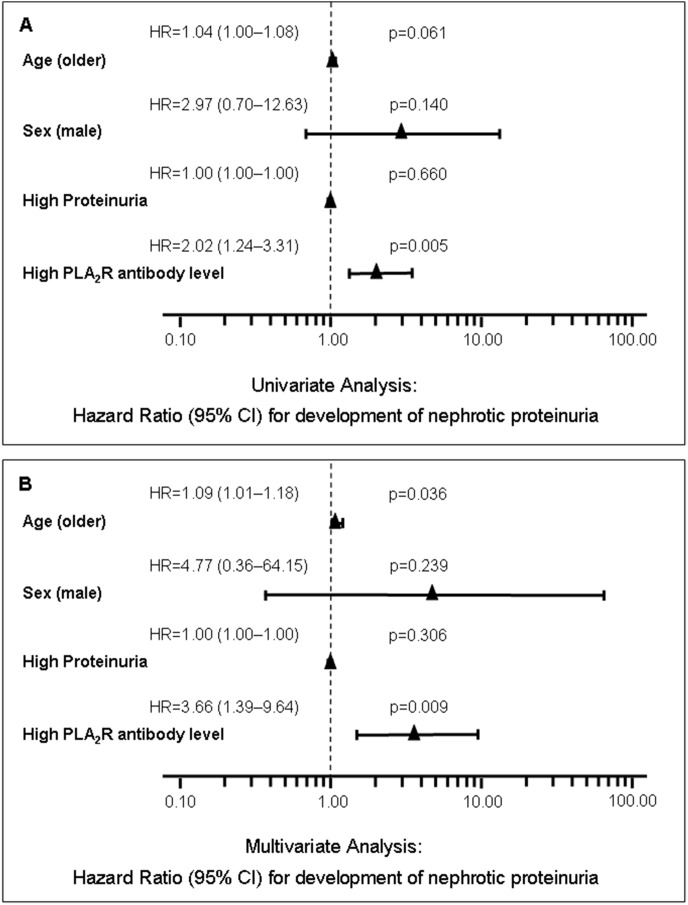
Hazard ratios with 95% confidence intervals and p values as estimated by univariate (A) and multivariate (B) Cox regression analysis. The explanatory variable PLA_2_R-Ab levels was ln-transformed prior to analysis. Effects of age and PLA_2_R-Ab levels are significant at α = 0.05. The corresponding hazard ratios indicate increasing risks for development of nephrotic range proteinuria with increasing age and PLA_2_R-Ab levels (high PLA_2_R-Ab levels).

**Figure 4:**
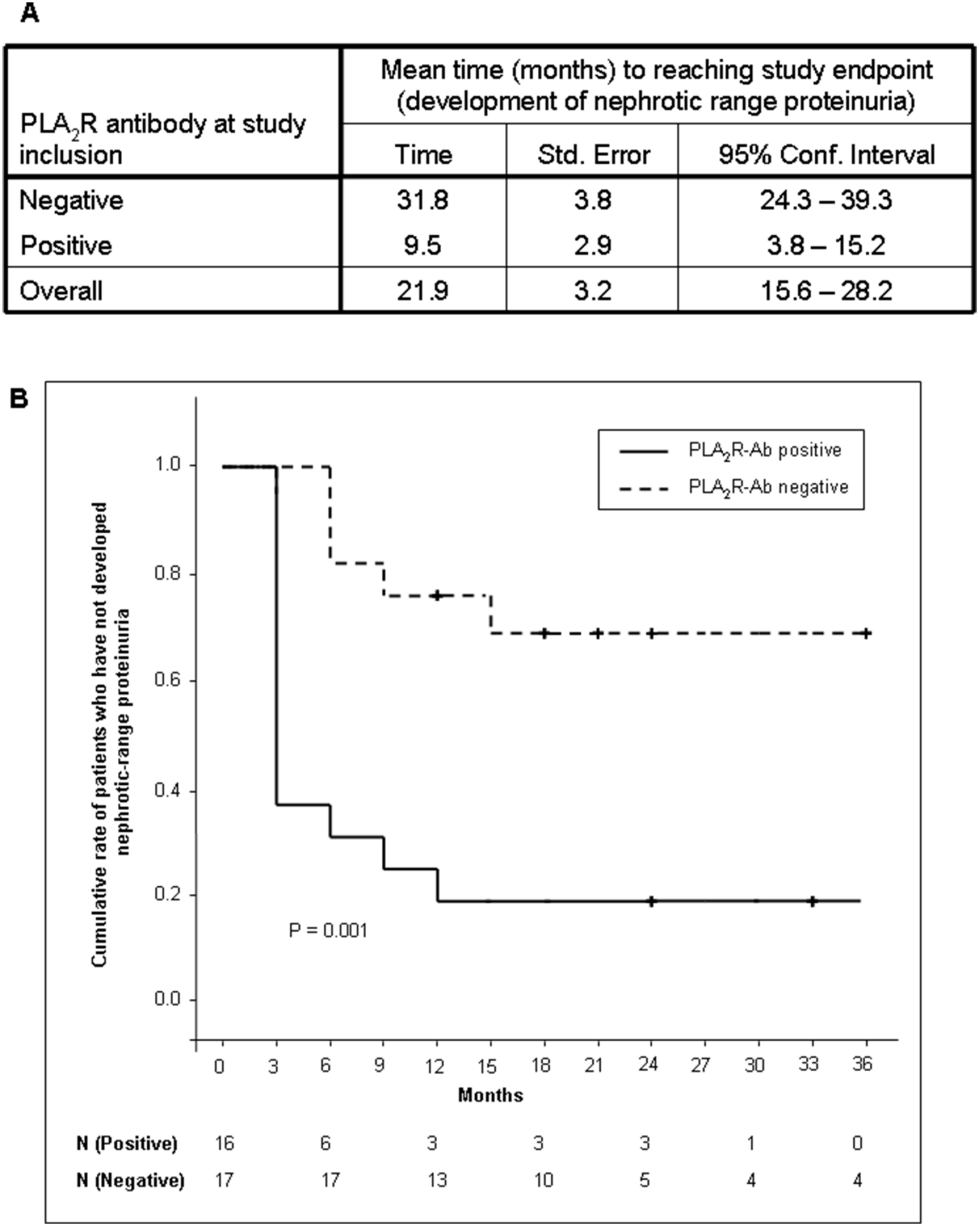
Survival analysis by PLA_2_R-Ab positivity. *4A) Time when PLA_2_R-Ab positive or negative patients developed nephrotic range proteinuria. 4B) Kaplan-Meier curves.* P values are from log rank tests. N = number of patients at risk at the different time points.

We performed a univariate and a multivariate Cox regression analysis to identify risk factors associated with the development of nephrotic range proteinuria in our patient cohort (Figure 3). The hazard ratios and their confidence intervals in the multivariate analysis indicate that on average, and adjusting for the effects of all other variables, the risk for developing nephrotic-range proteinuria increases by 9% with every year increase in age and by 266% per unit increase in the natural logarithm of the total IgG PLA_2_R-Ab level. PLA_2_R-Ab positive patients developed nephrotic proteinuria significantly faster than PLA_2_R-Ab negative patients (Figure 4).

### PLA_2_R-Ab levels, immunosuppression and remission of proteinuria during follow-up

PLA_2_R-Ab levels decreased in PLA_2_R-Ab positive patients during the study follow-up (Figure S1). During the follow-up 13 PLA_2_R-Ab positive patients developed nephrotic proteinuria and received immunosuppressive therapy (Figure 1). The PLA_2_R-Ab became negative in five of these patients, they all had a remission of proteinuria (three complete remission, two partial remission). The remaining eight patients remained PLA_2_R-Ab positive, only two of them had a remission of proteinuria (both partial remission). At the same time, five of them had a significant increase of serum creatinine during follow-up. Three PLA_2_R-Ab positive patients did not develop nephrotic proteinuria. They had the lowest PLA_2_R-Ab levels compared to the PLA_2_R-Ab positive patients. These patients reached a complete remission of proteinuria and became negative for PLA_2_R-Ab without immunosuppressive treatment.

Significantly more PLA_2_R-Ab positive patients received immunosuppressive therapy (13 out of 16) compared to PLA_2_R-Ab negative patients (two out of 17) (Fishers exact test: p<0.001). PLA_2_R-Ab positive patients received the following immunosuppressants: six patients received alkylating agents plus steroids, three of them were later switched to rituximab; one patient received alkylating agents plus steroids and was later switched to a calcineurin inhibitor plus steroids; two patients received calcineurin inhibitors and were later switched to rituximab; two patients received calcineurin inhibitors in combination with steroids, one of them was later switched to rituximab; two patients received rituximab. PLA_2_R-Ab negative patients received alkylating agents plus steroids, one of them was later switched to a calcineurin inhibitor plus steroids.

All PLA_2_R-Ab negative patients remained negative for PLA_2_R-Ab during the study follow-up. Nephrotic proteinuria developed in five PLA_2_R-Ab negative patients. Three of these patients achieved spontaneous remission of proteinuria (one complete remission, two partial remission), one patient had a partial remission under immunosuppressive therapy. The remaining patient had impaired renal function at study start (serum creatinine 2.76 mg/dl) and his proteinuria further increased. He went into end-stage renal disease 13 months after inclusion in the study. All other PLA_2_R-Ab negative patients, as well as all patients who became negative for PLA_2_R-Ab during the follow-up, had a stable serum creatinine. Eight patients remained positive for PLA_2_R-Ab throughout the study follow-up, five of them had a significant increase in serum creatinine. Two of these patients had a serum creatinine of 5.01 mg/dl and 5.32 mg/dl respectively (both 24 months after inclusion in the study). In these patients serum creatinine levels at study start were 1.7 mg/dl and 1.64 mg/dl respectively, showing a major decline in renal function during the follow-up.

When considering the PLA_2_R-Ab levels at the end of the study more PLA_2_R-Ab positive patients had no remission of proteinuria and had a significant increase of the serum creatinine during follow-up (six out of eight and five out of eight, respectively) compared to patients who were negative for PLA_2_R-Ab (one out of 25 for both parameters) (Fishers exact test: p<0.005 for both parameters).

## Discussion

Proteinuria is the hallmark of membranous nephropathy and it is used to assess the need for immunosuppressive treatment [1]. The outcome of patients with primary MN ranges from spontaneous remission to end-stage renal disease. Immunosuppressive treatment is beneficial concerning remission of proteinuria and maintenance of renal function, but associated with potential severe toxicity [7]–[10]. Since patients with high nephrotic range proteinuria have a worse clinical outcome [2], most clinical studies in patients with primary MN focussed on these patients. Only a few studies are available concerning patients with non-nephrotic proteinuria. These studies, however, show that patients with non-nephrotic range proteinuria do not always have an excellent prognosis. In some cases proteinuria increases and can be associated with bad clinical outcome [3], [11]. There are no clinical parameters, which predict whether non-nephrotic patients will develop nephrotic proteinuria. Clinical management of these patients depends on a watch and wait approach. Even if nephrotic proteinuria develops, it is difficult to decide whether immunosuppressive treatment should be started or not since some patients may still undergo spontaneous remission. The finding that PLA_2_R-Ab are present in most patients with primary MN and associated with disease activity was a major step forward in the clinical management of these patients [4], [5]. In this study, we prospectively analysed the development of proteinuria and clinical outcome in patients with MN and non-nephrotic proteinuria under treatment with inhibitors of the renin-angiotensin system, and assessed which potential role PLA_2_R-Ab might play for the clinical management of these patients.

Although all patients were non-nephrotic at study start, more than half of the patients developed nephrotic-range proteinuria during follow-up. This change resulted in the decision to treat most of these patients with immunosuppressants. Since the study was not blinded, it cannot be excluded that in some cases treatment decisions were influenced by the PLA_2_R-Ab levels, this however, was not part of the study protocol.

Some patients with MN, who were not nephrotic at the time of diagnosis, might experience a progression of their clinical disease activity and perhaps an unfavourable outcome. Nephrotic range proteinuria developed more frequently and faster in PLA_2_R-Ab positive patients compared to PLA_2_R-Ab negative patients. PLA_2_R-Ab levels were predictors for the development of nephrotic proteinuria in our patient cohort, showing a more unfavourable course of the disease. These patients were treated more often with immunosuppressants compared with PLA_2_R-Ab negative patients. At the end of the follow-up, less PLA_2_R-Ab positive patients experienced a remission of proteinuria and more PLA_2_R-Ab positive patients had a significant increase of the serum creatinine.

In addition to PLA_2_R-Ab, age was also significantly associated with development of nephrotic proteinuria in the multivariate Cox regression analysis. Male sex on the other hand showed an increased hazard ratio of 4.77 for the development of nephrotic proteinuria, but this was not statistically significant. One variable not analysed in this study was sodium excretion and sodium intake restriction, which might result in changing values of proteinuria over time particularly in patients on ACE inhibitors or angiotensin II receptor blockers.

A considerable part of the patients included in this study had low levels of proteinuria but high PLA_2_R-Ab levels. This observation confirms previous data showing that PLA_2_R-Ab levels are not directly correlated with proteinuria at a defined time [5], [12]. At the same time, this finding is very important, since serial PLA_2_R-Ab measurements allow to differentiate between two groups of patients: i) patients who were PLA_2_R-Ab positive and nephrotic, but are now undergoing remission and probably do not need immunosuppressive therapy from ii) patients who are PLA_2_R-Ab positive but the proteinuria is still sub-nephrotic and might eventually increase in the future. These patients would need special attention.

The clinical outcome of the three patients with an enhanced PLA_2_R staining in renal biopsy specimens and no detection of serum PLA_2_R-Ab suggests that these patients either had PLA_2_R-Ab before inclusion in the study, but were in immunological and clinical remission, or that these patients have an early phase of the disease where all antibodies (present in low levels) are bound in the kidney. The third option would be that a number of patients may have primary MN with circulating PLA_2_R-Ab but their levels are too low to be detected in the serum and can only be diagnosed by a renal biopsy [12].

Several studies have already shown a close association of changes in serum PLA_2_R-Ab levels and proteinuria over time [5], [13]. The same observation was made in the present study. Patients who remained positive for PLA_2_R-Ab at the end of the study, had fewer remissions of proteinuria and showed more often an increase in serum creatinine compared with patients who were negative for PLA_2_R-Ab. These data suggest that patients who become PLA_2_R-Ab negative, either spontaneously or under immunosuppressive treatment, have a better outcome than patients remaining positive for PLA_2_R-Ab.

A major open question which remains in the management of patients with primary MN is whether and how PLA_2_R-Ab levels could guide immunosuppressive treatment. Prospective, controlled studies will be necessary to show an eventual tight relationship of the PLA_2_R-Ab decrease and remission of proteinuria. Should such studies confirm that patients with non-nephrotic proteinuria and high PLA_2_R-Ab are in an active immunological status of the disease, it would suggest that these patients might benefit from early immunosuppressive treatment upon development of nephrotic proteinuria, without waiting and leaving them for over 6 months on high proteinuria.

In conclusion, our data show that the PLA_2_R-Ab status in patients with primary MN and non-nephrotic proteinuria at the time of diagnosis might be an important marker for the course of the disease.

## Supporting Information

Figure S1 **PLA_2_R-Ab levels during the study follow-up.** During the study follow-up PLA_2_R-Ab levels decreased in PLA_2_R-Ab positive patients (solid line). The bars show the SD-values of PLA_2_R-Ab levels for PLA_2_R-Ab positive patients (up) and PLA_2_R-Ab negative patients (down). “N” gives the number of patients for whom data were available for the times of follow-up. “§” shows a statistically significant difference (p<0.05) between PLA_2_R-Ab positive patients and PLA_2_R-Ab negative patients.
(TIF)
